# Cortical Folding Pattern and its Consistency Induced by Biological Growth

**DOI:** 10.1038/srep14477

**Published:** 2015-09-25

**Authors:** Mir Jalil Razavi, Tuo Zhang, Tianming Liu, Xianqiao Wang

**Affiliations:** 1 College of Engineering, University of Georgia, Athens, GA 30602, USA; 2 Cortical Architecture Imaging and Discovery Lab, Department of Computer Science and Bioimaging Research Center, University of Georgia, Athens, Athens, GA 30602, USA; 3 School of Automation and Brain Decoding Research Center, Northwestern Polytechnical University, Xi'an 710072, China

## Abstract

Cortical folding, characterized by convex gyri and concave sulci, has an intrinsic relationship to the brain’s functional organization. Understanding the mechanism of the brain’s convoluted patterns can provide useful clues into normal and pathological brain function. In this paper, the cortical folding phenomenon is interpreted both analytically and computationally, and, in some cases, the findings are validated with experimental observations. The living human brain is modeled as a soft structure with a growing outer cortex and inner core to investigate its developmental mechanism. Analytical interpretations of differential growth of the brain model provide preliminary insight into critical growth ratios for instability and crease formation of the developing brain. Since the analytical approach cannot predict the evolution of cortical complex convolution after instability, non-linear finite element models are employed to study the crease formation and secondary morphological folds of the developing brain. Results demonstrate that the growth ratio of the cortex to core of the brain, the initial thickness, and material properties of both cortex and core have great impacts on the morphological patterns of the developing brain. Lastly, we discuss why cortical folding is highly correlated and consistent by presenting an intriguing gyri-sulci formation comparison.

Brain development and related cerebral convolution have been fascinating research topics for more than a century^1^,^2^,^3^,^4^,^5^. The grooves in the convoluted brain are called sulci and the ridges between them are called gyri. The outer layer of the brain is composed of folded gray matter, called the cortex, which is made up of cell bodies and capillaries. The subcortex, or inner core, consists mostly of the white myelinated sheaths of neuronal axons. Human brain development involves a series of intricate and overlying processes, including neuronal precursor proliferation at the ventricular zone, neuroblast exodus from the ventricular zone, neuroblast migration, migration arrest, and neuronal organization^6^,^7^. During the development, the cerebral cortex experiences a noticeable expansion in volume and surface area accompanied by tremendous tissue folding^8^,^9^, which may be attributed to many possible factors, such as cranial constraint^2^, differential growth on the cellular base^10^, and axon maturation^11^. Despite decades of endeavors, the fundamental mechanism and key regulators of this crucial process remain incompletely understood^12^.

The most famous hypotheses in this area are related to the roles of internal tension in neuronal fibers (axons), differential expansion of the cortex, and radial growth^12^,^13^. In the internal tension hypothesis, axons exert a pulling force among cortical regions and thus induce folding^11^; however, there are many evidences against this hypothesis^14^. In the differential growth hypothesis, the outer layer of the brain grows at a faster rate than the inner layer, acting as the driving mechanism for cortical folding^15^,^16^,^17^. In most previous studies related to the elastic buckling models of the brain, the elastic modulus of the outer layer was higher than that of the core in order to produce buckling patterns which was not consistent with experimental observations^15^,^16^. In fact, the elastic modulus of the outer layer is not significantly different than that of the inner layer of the brain^18^,^19^,^20^. A computational model of cortical convolution^21^ suggested that without any additional assumption, the simple mechanical property of the cortex and differential growth are sufficient to produce cortical folding, which has been proven by other studies^13^,^17^.

Since cortical folding is a complicated phenomenon, computational modeling has begun to emerge as a powerful tool to validate or verify the results from experiments in addition to analytical models. For example, finite element (FE) analysis has offered valuable insight into the growth, morphology, and function of the brain. With FE models, it has been shown that a faster tangential cortical expansion leads to a shorter gyral wavelength, and that neither inner nor outer constraint (skull) is needed to produce folding^14^. Recently more 2D and 3D brain models have been implemented to clarify the role of mechanics during the brain development, and their results show that morphological abnormalities related to the developing brain can be presented by the mechanical models^22^,^23^,^24^.

Although significant progress has been made in recent years with respect to the modeling of the morphological evolution of the developing brain, there still remain many open questions which require additional experimental and analytical investigation. For example, why is the primary cortical convolution organization across subjects within each species highly correlated and consistent rather than random, and what factors count for this consistency as regulators? What is the contribution of glial cells and axons in the convolution process, and how can their roles be considered in the mechanical models? The aim of this paper is to develop an integrated analytical and computational tool to better model the growth and instability of the developing (cortex and core) brain, to investigate the criteria for instability and crease formation of the brain, and to link the instability to the geometrical and material properties of the brain. Finite element analyses are performed in order to leverage the results from the analytical approach and to predict the secondary morphological patterns of the developing brain. Finally, we will offer clues into the regulating mechanism of cortical folding by presenting an intriguing gyri-sulci formation comparison.

## Results

### Residual stress and instability induced by growth

For the proposed 2D cortex-core model, deformation and stress fields of a growing model can be derived (see details in the Methods section) based on the differential growth theory, incompressibility constraint, and the deformation gradient in the cylindrical coordinate system. Here, two cases of the growing brain model are considered: the first case is the isotropic growth for both cortex and core, and the second one is the tangential growth of the cortex but the isotropic growth for core. In the first case, we define 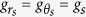 and 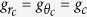 as the isotropic growth rate for the cortex and core, respectively. Here, 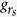 is the growth rate of the cortex in the radial direction, 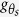 is the growth rate of the cortex in the circumferential direction, 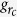 is the growth rate of the core in the radial direction, and 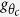 is the growth rate of the core in the circumferential direction. In the second case, the cortex of the brain model grows at a faster rate tangentially than the inner core. Here, the growth ratio of the cortex in the radial direction is considered as a unit, and the core experiences an isotropic growth with the growth rate *g*_*c*_. The growth rate of the cortex in the circumferential direction is assumed to be *g*_θ_. After a lengthy derivation, the radial and circumferential stress distribution in the cortex and core can be obtained.

**Figure 1:**
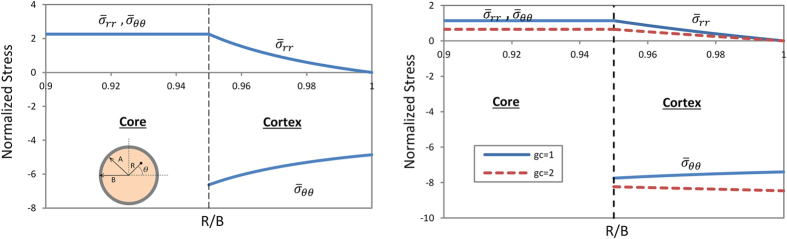
Normalized stress distribution in the radial and circumferential directions for the growing cortex-core model; (**a**) isotropic growth of the cortex and core, *g*_*s*_/*g*_*c*_ = 3 and *A*/*B* = 0.95; (**b**) circumferential growth of the cortex and isotropic growth of the core, *g*_*θ*_/*g*_*c*_ = 3 and *A*/*B* = 0.95.

Figure 1 depicts the normalized radial 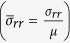 and circumferential 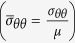 Cauchy stresses for both cases of the growing brain model. Here, it is considered that the shear moduli (*μ*) of the cortex and core of the brain are the same^24^. The ratio of the initial inner to outer radius of the cortex is *A*/*B* = 0.95, for the definition of *A*, *B* and *R* please see the Methods section. In both cases, the cortex grows three times faster than the core, which means *g*_*s*_/*g*_*c*_ = 3 or *g*_*θ*_/*g*_*c*_ = 3 (the number 3 is chosen here to show the stress distribution pattern, more discussion about the growth ratio will be presented in the following section).

Figure 1 also shows that, in both cases, the mismatch between the growth rates of the cortex and core leads to the appearance of residual stresses in the structure. It is noticed that both radial and circumferential stresses in the core are tensile. However, the radial stress in the cortex is tensile but the circumferential stress is compressive, which is consistent with experimental observations^14^,^25^. Also, the core of the brain model is under a homogeneous stress state, and its magnitude is equal to that of the radial stress of the cortex at the interface, 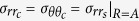. For the purpose of illustration, here only the range of the core from *R*/*B* = 0.9 to *R*/*B* = 0.95 is shown in the plot. The compressive stress in the cortex may have an important effect on the onset of instability. The stress state of the core is independent of its shear modulus and is homogenous in all growth ratios. Interestingly, for the case of isotropic growth (Fig. 1a), the magnitude of the normalized stresses is just a function of the growth ratio of the cortex to core and is not related to the individual growth rates of the cortex and core. For the case of tangential differential growth (Fig. 1b), the growth rate of the cortex in the radial direction is considered as a unit. In contrast to the isotropic growth of the cortex, the change of the growth rate of the core modifies the stress distribution of the brain model while keeping the growth ratio of the cortex to core unchanged. The magnitude of the radial stress in the case of the tangential growth is lower than the one in the isotropic case in general, but the magnitude of the circumferential stress is higher. For both isotropic and tangential growth cases, when the cortex grows faster than the core, larger compressive stresses always occur in the outer layer of the cortex. These kinds of compressive stresses in the surface of soft materials engenders instability and leads to the formation of creases^24^,^26^.

Hence, in order to find the critical growth ratio which makes the brain model lose stability, we follow Suo’s work^26^ to derive the formula for both cases below (for more details please see instability analysis subsection in the Methods section)


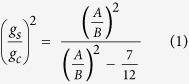



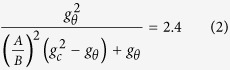


**Figure 2:**
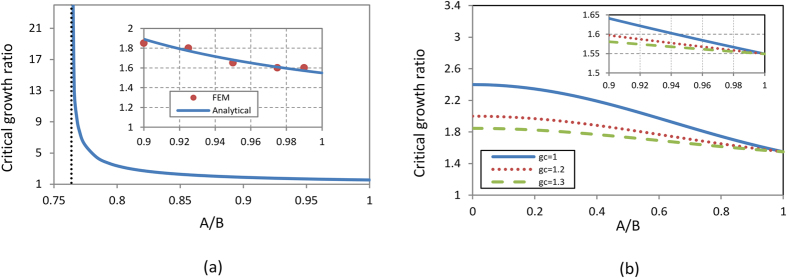
Critical growth ratio of the brain model to trigger instability: (**a**) Isotropic growth of both the cortex and core; (**b**) Tangential growth of the cortex and isotropic growth of the core. In both cases, the cortex and core have the same material properties.

From the equations, it clearly shows that the critical growth ratio depends on the initial configuration of the model. Similar to the stress distribution in the isotropic growth case, here the critical growth ratio (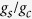) is not related to the absolute value of the individual growth rates for either the cortex or the core. For the case of the tangential growth, it can be observed from Eq. (2) that the critical growth ratio, as defined by 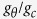, is not just a function of geometric parameters, but also depends on the absolute value of the cortex or core growth rate. Figure 2 plots the critical growth ratio for the onset of creases in the brain model as a function of geometric parameters and growth rates of the core for both cases.

Figure 2a shows that below a critical value of 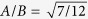, instability will not occur no matter what the growth ratio of the cortex to core is. In other words, a brain model with a thick cortex is more stable than one with a thin cortex during the growing process. With the decrease of the cortex thickness, the critical growth ratio for instability or creasing decreases and 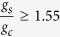 is required to start instability for a model with very thin cortex. Here, the geometry of the cortex as *A*/*B* = 0.95 in the brain model is considered^27^. Based on Fig. 2a, the critical growth ratio of the cortex to core for crease formation is around 1.68, which means from the analytical viewpoint, the cortex should grow 1.68 times faster than the core to generate cortical folding. In Fig. 2b for the tangential growth of the cortex, the instability always happens in the brain model once it reaches the critical growth ratio irrespective of the thickness ratio of the cortex to core. The general trend is that by decreasing the thickness of the cortex in the model, the critical growth ratio for instability also decreases. On the other hand, by increasing the growth rate of the core, the critical growth ratio of the cortex to core for instability will decrease, indicating that a brain model with a fast growth in the core easily loses stability. This trend is more pronounced when a brain model with a thick cortex is considered. The solid line in Fig. 2b refers to a situation where the core does not grow (*g*_*c*_ = 1) and it acts as a rigid substrate. In the model with a thin cortex, as indicated in the subplot of the Fig. 2b, the critical growth ratios are quite close to each other regardless of the core growth rate. The critical growth ratio of instability for the model with a very thin cortex is close to 1.55, which is the same as the value from the case with the isotropic growth. By comparing both isotropic and tangential growth cases under the same thickness of the cortex, it is found that the critical growth ratio of instability for the isotropic growth case is higher than that of the tangential growth case. This can be attributed to the increase in the thickness of the cortex due to the growth in the radial direction, which exerts a positive effect on the stability of the brain model.

### Crease formation and post-perturbation

**Figure 3:**
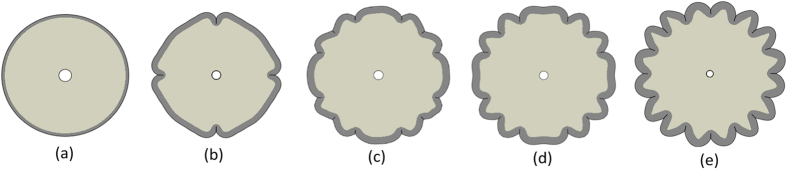
Evolution of crease formation in a growing brain model with *A*/*B* = 0.95 and *g*_*s*_/*g*_*c*_ = 3; **(a)** *g*_*t*_ = 1; **(b)** *g*_*t*_ = 1.408; **(c)** *g*_*t*_ = 1.462; **(d)** *g*_*t*_ = 1.509; **(e)** *g*_*t*_ = 1.691 Figures are not in the same scale. For the definition of *g*_*t*_ please see the Methods section.

When the growth ratio of the cortex to core in the brain model is beyond the critical value, the system starts to lose stability and form creases in the outer surface of the model. This happens in order to release the elastic energy in the brain model partly and therefore reach another stable configuration. Since the analytical solution cannot predict crease formation after the critical growth ratio, FE models are implemented to predict folding patterns after instability. Figure 3 shows a morphogenesis evolution of a growing brain model under a series of growth ratios. At the beginning, the number of the creases in the model is few and the depth of the creases is shallow. With the continuing growth, more creases occur on the surface of the outer cortex. It is clearly noticed that the cortical layer in the gyri region is significantly thicker than the one in the sulci region, which is highly consistent with experimental observations^28^. Analytical and computational results for the critical growth ratio for instability and crease formation are also in good agreement as shown in Fig. 2a.

**Figure 4:**
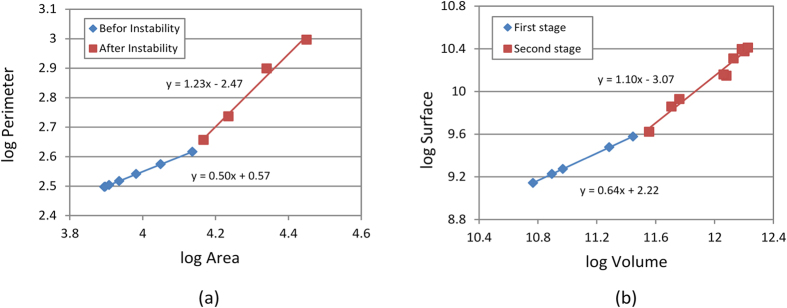
(**a**) Variation of the cortical perimeter versus area in the brain model with ***A*****/*****B***** = 0.95** and ***g***_***s***_**/*****g***_***c***_** = 3** before and after developing convolutions (**b**) Cortical surface area versus volume of a spatio-temporal atlas of developing brains ranging from 23 to 37 weeks gestational age (GA)^63^. Brain volume was measured directly on the volumetric atlas. Cortical area was measured on triangular mesh cortical surfaces reconstructed from the volumetric atlas via marching cube based methods^64^. The age range of the blue dots is 22 weeks to 27 weeks while that of the scarlet dots is 28 weeks to 37 weeks.

Therefore, these preliminary findings may offer clues to characterize and evaluate some specifications of a developing brain. For instance, one remarkably distinctive feature of the mammalian brain is the unique relationship between the cortical surface area and the brain volume during the morphological evolution process. If the cortical surface area were to increase in a purely geometric fashion, then its increase would follow the two-thirds power relationship of the brain volume^29^,^30^. During the actual brain development, however, gyrification allows for an increasingly large cortical surface. Therefore, the surface area of the cerebral cortex increases almost as the first power (*α* = 0.90) of the brain volume^29^. Figure 4a shows the variation of the perimeter of the cortical layer as a function of the total area of the model. In the plane-strain 2D model, the perimeter of the cortical layer represents the cortical surface area, and the area of the model represents the total brain volume as a 3D model does. Cortical folding maximizes the surface-to-volume ratio of the brain to increase the number of nuclei and decrease the relative distance between them.

Before instability and crease formation, the perimeter of the cortical layer is expected to vary as the square root of the area of the model, as indicated in Fig. 4a with blue, because the growing brain model keeps a circular shape. However, when the brain model develops convolutions, the change of the perimeter of the cortical layer deviates from the previous trend related to the growth of the total area, and the perimeter of the cortical layer grows faster than expected. Figure 4b shows the relationship between the surface area and volume of a real developing brain during the gestation period. It is clear that there is a good agreement between the computational and experimental results. For the real brain, during the first stage before crease, the slope is somehow greater than the one from computational results. This can be explained that in the simulation the brain is modeled as a 2D circle while in reality this is not the case.

### Effect of cortex thickness and material property

**Figure 5:**
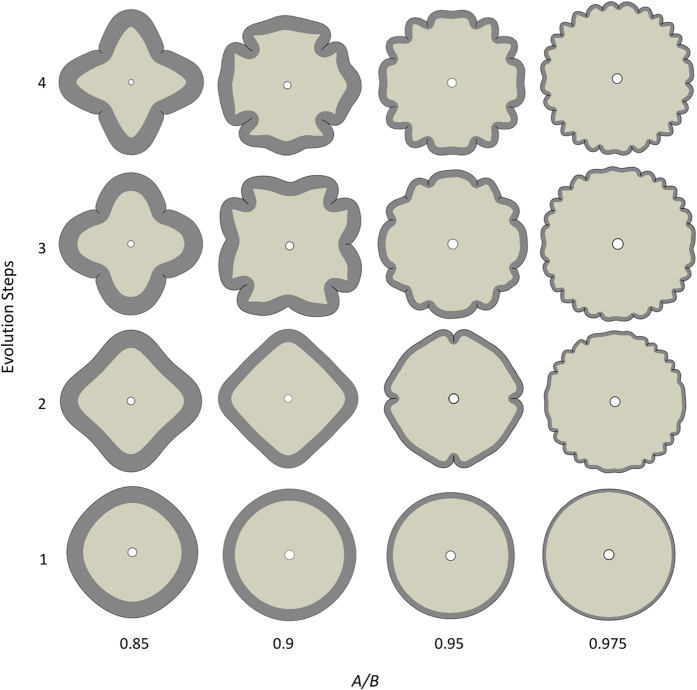
Morphological evolution of a growing model with different cortex thickness under μ_*s*_/μ_*c*_ = 1 and *g*_*s*_/*g*_*c*_ = 3; time step from 1 to 4 shows the evolution of morphology of the models (figures are not in the same scale).

It was found in Fig. 2 that the thickness of the cortex is a crucial parameter in determining the critical growth ratio for instability in a growing brain model. Therefore, it is worthwhile to investigate the effect of the cortex thickness on the morphological pattern of the brain after instability. Here, several models with different thickness for the cortex have been carried out as depicted in Fig. 5. As observed in the analytical section, a large critical growth ratio for starting instability in the model with a thick cortex is needed. As shown in the first column (*A*/*B* = 0.85) of Fig. 5, the number of creases is only four and the evolution of the creases is very stable without any increase in the number of creases. This phenomenon is experimentally observed in Lissencephaly, a malformation of the brain with a thick cortex and fewer gyri and sulci^6^,^31^, see Fig. 1 of the reference32. For the purpose of comparison, we consider a brain model with a thin cortex (*A*/*B* = 0.975) to illustrate the change of brain morphology due to the change of the thickness of the cortex.

The last column of Fig. 5 shows the formation of numerous small gyri and sulci in the brain model after instability compared with a normal one in Fig. 3. The morphological pattern has been experimentally observed in polymicrogyria malformation, where the surface of the brain normally has many folds and the cortex thickness is smaller than the one in a healthy brain. Either the whole surface (general) or parts of the surface (local) can be affected^6^,^33^, see Fig. 2 in the reference^34^.

In addition to the geometrical parameters, the material property of the brain model may also play a vital role in the convolution pattern of the brain. Hence, in what follows, the effect of material property of the brain will be investigated. Before that, however, it is noteworthy to mention that it is still very difficult to characterize the brain mechanical property accurately because characterization of the brain tissue highly depends on the definitions, tools and procedures used^18^. For example, it has been shown that the brain exhibits an anisotropic behavior; additionally, the white matter has more heterogeneity than the gray matter^19^,^35^,^36^. The shear moduli of the grey and white matter have been reported differently in various studies^22^,^23^,^37^,^38^. Therefore, there is no firm and proven data for the material properties of the grey and white matter. Here, the focus is on the morphological evolution of the brain with respect to the shear moduli ratio of the cortex to core rather than the absolute magnitudes for both parts.

**Figure 6:**
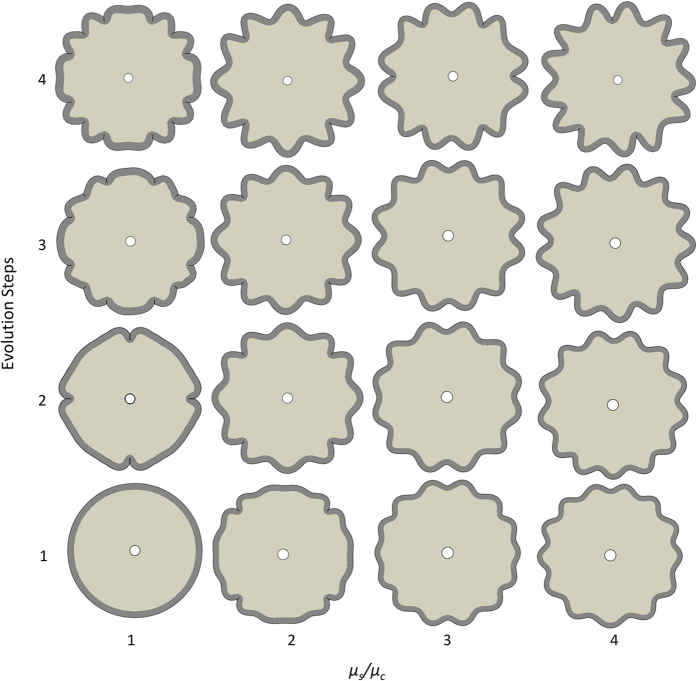
Morphological evolution of a growing brain model with different shear modulus ratios of the cortex and core under *A*/*B* = 0.95 and *g*_*s*_/*g*_*c*_ = 3; time step from 1 to 4 shows the evolution of morphology of the model (figures are not in the same scale).

Figure 6 shows the morphological evolution of a growing brain model with different material properties of the cortex and the core under the same geometric configuration, *A*/*B* = 0.95, in which the shear moduli ratio of the cortex to core (μ_*s*_/μ_*c*_) varies from 1 to 4. The brain model with a small shear moduli ratio prefers to develop creases first after instability; however, when the shear moduli ratio is large, the brain model prefers to wrinkle first and then develop creases. This finding reveals that, for the formation of creases in the brain, the shear moduli of the cortex and the core should be close to each other, which has been experimentally validated in the recent work^39^. It also reveals that the number of wrinkles depends on both the shear moduli ratio and the thickness of the cortex in the brain model^40^. These findings demonstrate that the shear moduli ratio of the cortex and the core exerts a great impact on the morphological pattern of the growing brain model after instability. It also implies that a change of the stiffness in the cortex or core of the brain caused by abnormalities or disorders may lead to a change in the pattern of the formation of gyri and sulci.

## Discussions

After instability occurs in a growing brain model, a thick cortex leads to the formation of fewer gyri and sulci (low gyrification index). This is consistent with Lissencephaly malformation in the developing brain. In contrast, a thin cortex leads to a high number of shallow gyri which is an abnormality referred to as Polymicrogyria^23^.

**Figure 7:**
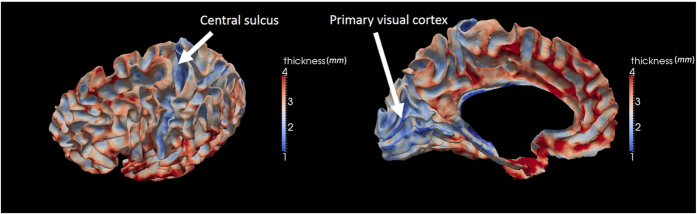
The thickness of an adult cerebral cortex mapped onto its white matter triangular mesh surface. White arrows highlight locations of central sulcus and primary visual cortex where thinner cortices are found. Figures are constructed based on the data from the Human Connectome Project (http://www.humanconnectome.org/)^65^ and by using the FreeSurfer toolkit^28^,^66^.

Evidence which may prove that a thinner cortex leads to more creases in a brain is the central sulcus, a primary somatosensory cortex which roughly consists of Brodmann areas #1, 2 and 3^41^. The central sulcus and the primary visual cortex are among the thinnest parts in the cortical region of the human brain^28^. From the imaging data, it can be observed that the central sulcus is one of the deepest sulci in the brain and that the primary visual cortex has the most complicated folding patterns among all cortical cortex regions^5^,^42^,^43^ as shown in Fig. 7. This lends compelling credence to the statement that a thinner cortex leads to more creases in cerebral cortex.

**Figure 8:**
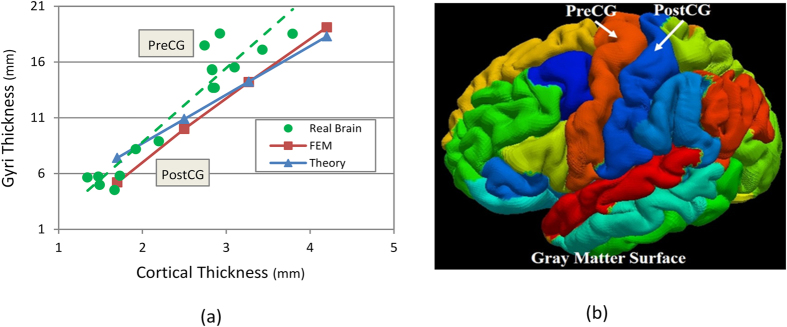
(**a**) Dependency of gyri thickness on the thickness of the cortex. Gyral thickness is measured on gray matter (cortex) surfaces. (**b**) Gyri annotation on adult brain gray matter surfaces, PreCG: pre-central gyrus; PostCG: post-central gyrus. Picture (**b**) is constructed based on the data from the Human Connectome Project (http://www.humanconnectome.org/)^65^. The HCP MRI data pre-processing pipelines are primarily built by using tools from FSL and FreeSurfer^67^,^68^,^69^.

In addition to the quantitative analysis of the surface-to-volume ratio of a developing brain, there is another quantitative index crucial to interpreting the formation of creases in brain: the cortical thickness. Figure 8a shows the linear relationship between the thickness of gyri and the cortical thickness in the brain model. This dependency and relationship can also be observed in the different parts of a real brain as indicated in Fig. 8b.

From Fig. 8a, it can be inferred that the thickness of gyri is closely related to the thickness of the cortex. Given that gyri thickness in the FE brain model is roughly calculated and compared with the wave length of a buckling stiff layer on a soft substrate, a similar trend is observable from an analytical viewpoint. The wavelength *λ* of a wrinkling pattern in the film-substrate model predicted by the linear buckling theory is 
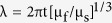
, where *t* is the thickness of the film and μ_f_ and μ_s_ are the shear moduli of the film and substrate respectively^44^. Here, as mentioned before, the shear moduli for the cortex and the core are the same. From the analytical formulation, it can be seen that there is a linear relationship between wavelength (in the FE models referred as gyri thickness) and the thickness of the film, which is the same as the result from the FE simulations in this study^45^.

**Figure 9:**
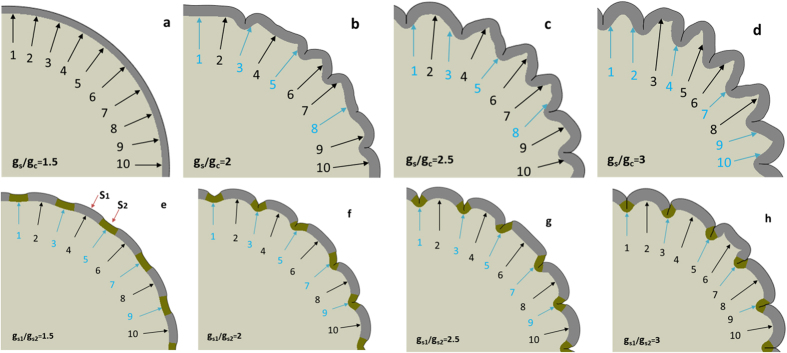
Cortical convolution patterns with different growth speeds (*g*_*s*_/*g*_*c*_). Numbered arrows indicate the corresponding locations on the cortex. In the first row (**a**–**d**), no growth difference is set to the cortex while in the second row (**e**–**h**) the cortex regions highlighted by the even numbered arrows grow faster than those highlighted by the odd numbered arrows 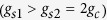. Based on observation, blue arrows are used to suggest sulcal regions and black arrows to suggest gyral ones. The simulation results shown in each sub-figure occur at the same simulation time.

As mentioned in the introduction section, there is an intriguing question to answer: why is the primary cortical convolution organization across subjects within each species highly correlated and consistent rather than random? Here, we would like to offer some preliminary data to initiate the discussion as to what factors determine these correlated patterns in the brain. It was reported that radial glial cells (RGCs) with lower levels of Trnp1 could generate basal progenitors (BPs), also known as intermediate progenitors (IPCs), and basal radial glial cells (bRGCs), which eventually contribute to generate neurons^46^,^47^,^48^. More interestingly, Trnp1 levels exhibit regional differences in the cerebral cortex of human fetuses^47^. Therefore, here we hypothesize that convolution patterns might stem from the heterogeneous regional growth rates. Convex patterns may be produced in locations where the cortical plate grows faster because more neurons migrate towards those regions. It had been reported that radial/tangential heterogeneous growth rate among laminas might be a critical factor in generating convolution^10^,^16^,^49^. Therefore, we reproduce this factor in our model by setting the growth speed of the cortex (*g*_*s*_) faster than that of the core (*g*_*c*_). The upper row of Fig. 9 shows the results with a variety of growth ratios (*g*_*s*/_*g*_*c*_) between the cortex and core of the brain model. Comparing with previous results, it is observed that convolution becomes more elaborate with the increasing growth ratio (*g*_*s*/_*g*_*c*_), but the growth ratio does not regulate the convolution patterns. For example, 10 corresponding locations have been highlighted by numbered arrows on the cortex. With different growth ratios for the cortex and core of the model, consistent folding patterns are not always reproduced. To be more specific, the positions on the cortex are blue if they are located near the ‘sulcal roots’ and black otherwise. In Fig. 9b,c, arrow #2 appears in gyral regions; while it is in the sulcal root in Fig. 9d. Another example is the location of arrow #8, which appeared on gyral wall in Fig. 9c, and on the gyri in Fig. 9d. In contrast, based on the same model, we introduce a regional growth speed difference within the cortex (the second row of Fig. 9. Under the assumption that regions with more RGCs accumulate more migration neurons to the cortical plate, we initialize the cortex by assigning certain periodic cortex regions (*s*_1_, highlighted by black arrows) higher growth speeds than the others (*s*_2_, highlighted by blue arrows), i.e., 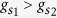. In order to simplify the analysis, the growth speed of the *s*_2_ region is set as twice that of the core, i.e., 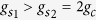. It is interesting to see that convex and concave patterns are consistently formed in *s*_1_ and *s*_2_ regions, respectively. These simulation results suggest that the cortex-core differential growth assumption may only produce unregulated convolution while consistent and reproducible convolution patterns on the cerebral cortex are regulated by regional growth heterogeneity. This is controlled by regional differentiation of RGCs in the early stage of development in the fetal brain.

Last but not the least, it is worthwhile to mention that in the application of analytical and computational models there are some simplifications and assumptions which impose limitations to the results. In our model, the cortex and the core of the brain are assumed to be isotropic while in the real brain both grey and white matter show anisotropic properties^35^,^36^. For example, the anisotropy of the core (white matter) has been modeled by a stretch driven property to mimic the axons contribution to the deformation of the developing brain^17^,^23^, while the assumption cannot exactly cover the role of the axons and glial cells on the regulation of convolution patterns in a developing cerebral cortex^46^,^47^,^50^,^51^. This still requires extensive research in order to consider appropriate glial and axonal contributions in the mechanical models. In this paper and other studies, smooth circular or elliptical initial shapes have been considered as the initial geometry of a developing brain^17^,^22^, while a real developing brain at the early stage is not in a regular shape^52^. Therefore, modeling the brain with a proper initial geometry may lead to a better interpretation of the convolution pattern of the brain. Finally, most current models for cerebral convolution in either human or other animals, e.g. ferret, are two dimensional models. A 3D model is obviously more realistic since brain convolution is a three dimensional dynamic process. Also, the gyrification index from the 2D model is less accurate than what it is in the real brain, where it can be up to 3^3^. Therefore, the usage of a 3D brain model will find a promising way to present the spatial convolution pattern of the developing brain; this view will be explored in our future work.

## Conclusions

In this paper, we have explored the morphological evolution and malformation mechanism of a developing brain in the fetal stage due to the biological growth from a mechanical viewpoint. An integrated analytical and computational tool is implemented to determine both the stress distribution and the critical growth ratio for instability of the brain model. Results show that in both isotropic and tangential growth of the cortex, after a critical point, the model prefers to destabilize and releases the potential energy partly via crease formation to reach another stable configuration^53^. Both analytical and computational findings are in good agreement with previous results for the differential growth hypothesis^12^.

After instability, a thick cortex in the brain model leads to the formation of fewer gyri and sulci (low gyrification index). This is consistent with Lissencephaly malformation in a developing brain. In contrast, a thin cortex leads to a high number of shallow gyri which is referred to as Polymicrogyria abnormality^23^. With respect to the effect of material property of the brain model, results demonstrate that the shear moduli ratio of the cortex to the core plays a crucial role on the morphological evolution of a developing brain^21^,^22^. High cortex stiffness causes the growing brain model to preferentially wrinkle instead of creasing. However, in reality the brain develops creases; this insinuates that there is no big difference in material property between the cortex and the core of the brain. It was also found that the cortex-core differential growth assumption may only produce unregulated convolution while consistent and reproducible convolution pattern on cerebral cortex is regulated by regional growth heterogeneity. Finally we hope that our study can stimulate more interests in this field, therefore opening new windows towards a better understanding of brain disorders and malformations.

## Methods

Generally, to determine the critical growth ratio for the onset of folding in a developing brain model, we need to analyze the stability of a mathematical model. However, analytical method cannot predict the evolution of complex cortical convolution after the critical point. Therefore, following the critical growth ratio of the brain model, non-linear finite element models with finite growth assumption are employed to present crease formation and the secondary morphological folds of the growing brain. Here, the concepts of each approach adopted in this paper are briefly introduced.

### Analytical Method

**Figure 10:**
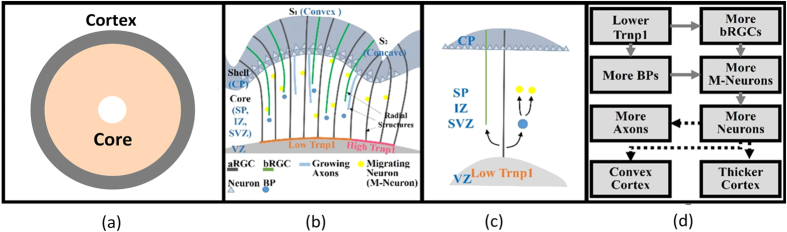
(**a**) An idealized 2D model of the brain; (**b**,**c**) biological basis of neurogenesis that is of interest; (**d**) flow chart of how Trnp1 regulates the cortical folding patterns. The dashed line arrows suggest macro-scale features of the cortex. Abbreviations: aRGC, apical RGC; bRGC, basal RGC; BP, basal progenitor; CP, cortical plate; VZ, ventricular zone; SVZ, subventricular zone; SP, subplate; IZ, intermediate zone.

A two-dimensional (2D) circular model consisting of two-layer soft tissue (Fig. 10) is constructed to investigate the mechanism of cortical folding in the first stage. The shell of the model (cortex) represents the developing cortical plate while the core is a simple organization of the subplate, intermediate zone and ventricular zone. The cerebral cortex is a thin (2–4 mm)^12^ layer in contrast to the inner core which, here, is modeled with a radius of 50 mm. As mentioned in the introduction, for the differential growth hypothesis, the outer layer grows at a faster rate than the inner layer of the brain, which is considered as the driving mechanism of cortical folding. Therefore, we consider the outer layer of our model (cortex) grows faster than the inner layer (core).

Figure 10 represents the biological foundation of our brain model. We use a flowchart in Fig. 10d to summarize the model. Generally, RGCs with lower levels of Trnp1 could generate basal progenitors (BPs), also known as intermediate progenitors (IPCs), and basal radial glial cells (bRGCs). BPs will produce neurons while bRGCs provide additional guiding structures inducing faster neuron migration and finally resulting in considerable radial and lateral expansion, i.e. the convex folding pattern suggested in refs 47, 48. Therefore, at the cellular level, the distribution difference of RGC regulates radial expansion of the cortical plate by controlling the amount of migrating neurons. Interestingly, this regional difference can be found in the cerebral cortex of human fetuses^47^.

#### Basic equations for a growth model

**Figure 11:**
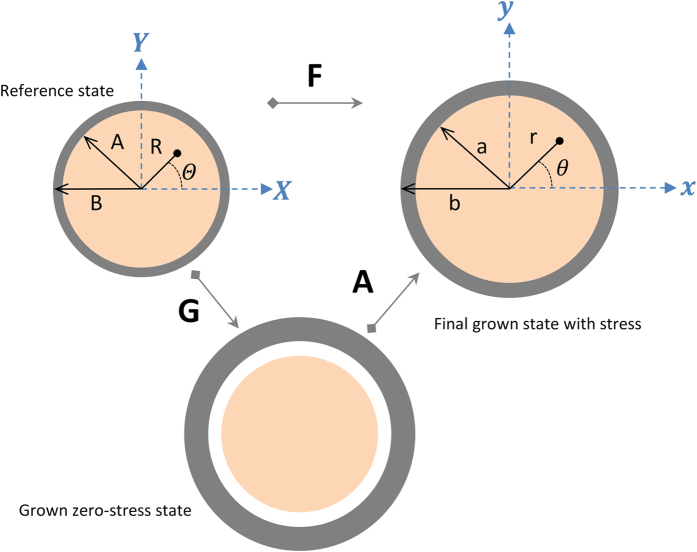
Initial and current states of a growing brain (cortex-core) model.

Here we consider the human brain as a living system with a growing outer cortex and inner core as shown in Fig. 11. Any point 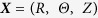 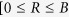, 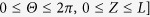 in the reference state and before growth is mapped by transformation to the final state while after growth, 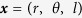 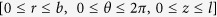, is mapped in the current state. Following the theory of multiplicative decomposition, the deformation gradient, ***F***(***X***), is decomposed to a growth tensor, ***G***(***X***), indicating the addition of materials and an elastic deformation tensor, ***A***(***X***), which describes pure deformation resulting from stress^54^.

The growth tensor maps the stress-free reference configuration to a grown stress-free state, and then the elastic deformation tensor maps the grown state to a stressed and final current state. Deformation gradient ***F*** maps the tissue from the stress free state before the growth to the stressed state after the growth.


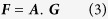


where 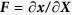. While both ***G*** and ***A*** tensors may be incompatible deformations, their multiplication, ***F***, should be a compatible deformation. In general, the elastic deformation of living soft tissues yields little volume change; therefore, the nonlinear response of these materials can be described by an isotropic incompressible hyperelastic material. The incompressibility implies that the determinant of the elastic deformation tensor should be equal to unit, i.e. det ***A*** = 1. Generally, the growth tensor depends on the stress state and deformation, as well as other factors. For simplicity, it is assumed that the growth process with a known spatial distribution, insinuating that all of the biological information is independent of stresses^55^. Due to the growth, this cortex-core structure deforms axisymmetrically; therefore, the deformation field after growth is just a function of the radius, *r* = *r*(*R*). Also, in order to eliminate longitudinal effects and focus on the study of in-plane bifurcation, the plane-strain assumption is considered here^17^,^23^. Many biological soft tissues can be modeled by a hyperelastic material with a strain energy function *W*(***A***). Therefore, the Cauchy stress ***σ*** is related to the strain energy function by^55^


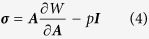


where *p* is the hydrostatic pressure and ***I*** is a second-order unit vector. In the absence of any body force, mechanical equilibrium imposes


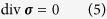


where “div” stands for the divergence operator in the current configuration. There are several proposed material behaviors for hyperelastic material; here a simple and common model, isotropic nonlinear neo-Hookean, is implemented.


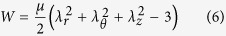


where 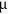 is the shear modulus and 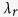, 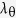 and 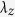 are the radial, circumferential and axial principal stretches, respectively.

#### Instability analysis

**Figure 12:**
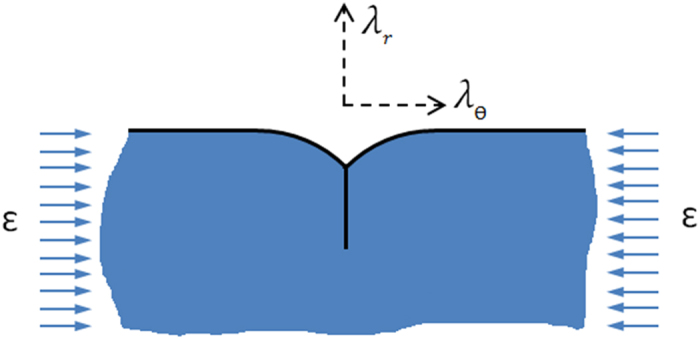
Crease formation on the surface of a soft material under compressive strain, ε. For the formation of a crease, applied compressive strain should be beyond the critical strain.

Creases usually occur at the surface of soft materials without hard skins when an initially smooth surface forms a self-contacting shape with a sharp ridge or sulci^56^ as shown in Fig. 12.

According to Fig. 12, the critical condition for the onset of crease formation for a compressed neo-Hookean soft material in the circumferential direction (normal to radial direction) is that the ratio of the principal stretch in the radial direction to the circumferential direction in the outer layer should be more than 2.4, *λ*_*r*_/*λ*_*θ*_ ≥ 2.4. This relation was derived by comparing the elastic energy in a creased body with that in a smooth body^26^ and has been used to predict the critical growth ratios for instability in a growing soft matter with a confined boundary^57^,^58^. The compressive strain can be generated either by the external stimuli or growth in the confined boundary. In this model, since the cortex grows faster than the core, the core acts as a confinement to the cortex. Due to growth mismatch between the cortex and the core, compressive strain is induced on the free surface of the cortex (see Results section). When it exceeds the critical value, creases are developed on the surface of the cortex. Following this critical value of the onset of crease formation in soft materials and the assumption of the plane-strain condition (*λ*_*z*_ = 1 and *g*_*z*_ = 1), the critical growth ratios for triggering instability and generating creasing in our model are determined.

### Numerical Method

To predict realistic cortical morphologies after the onset of instability in the growing brain model, a computational model based on non-linear finite element with isotropic growth in both the cortex and core of the brain model is carried out. The plane-strain models with neo-Hookean material behavior for both the cortex and core are performed, and brain growth is simulated via thermal expansion^57^,^59^. The outer cortex of the brain model is allowed to be self-contact. In order to apply a fixed boundary in the model, a small hole around the center of the core is placed. This consideration makes it easy to adjust structured (mapped) mesh to the model. Since this fixed boundary is far enough from the crease formation sites, its effect on the deformation patterns can be negligible. Dynamic-Explicit solver in the commercial software Abaqus (version 6.13-4)^60^, which is suitable for large deformation, nonlinear and quasi-static problems, is implemented to perform the secondary morphological changes in the brain model. Both the cortex and core sections of the brain model are meshed by a plain-strain, linear CPER4 element type with linear and quadratic viscosity of 0.06 and 1.2, respectively. To ensure the robustness of the simulation results, a variety of different meshes have been employed to investigate the folding patterns. For simplicity, we do not include the mesh-independent results here. In our dynamic model, the inertial force acts as the perturbation trigger for instability. Deformation patterns after instability are not guaranteed to be exactly symmetric although the initial model is symmetric^61^,^62^. Robustness studies conclude that as long as the mesh size is small enough, the qualitative features of our model do not depend on mesh size. The patterns of the brain model after growth do not depend on the absolute amount of shear modulus of the cortex and core and they just depend on their modulus ratio. With the condition of incompressibility and isotropic growth in the brain model, the overall growth ratio of the brain model (*g*_t_) can be defined as the surface ratio of the deformed area *S* to the initial area *S*_0_ of the 2D brain model, 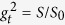.

**Figure 13:**
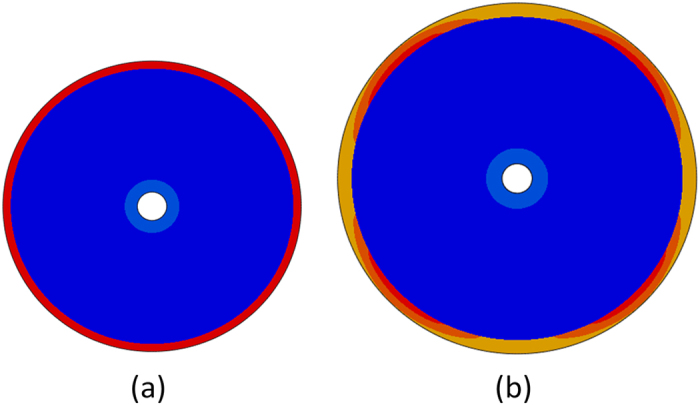
A growing brain model with von Mises stress distribution at two different growth ratios (**a**) *g*_*t*_ = 1.014, (**b**) *g*_*t*_ = 1.194. The cortex grows three times faster than the core, 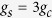 (3 is an arbitrary number). The shear moduli for both the cortex and core of the model are same. Blue to red indicates stress distribution.

Figure 13 shows a growing cortex-core model at two different growth ratios before the initiation of instability. After a small amount of growth, the von Mises stress distribution is uniform in both the cortex and the core, Fig. 13(a); however, with the increase of the growth ratio, this uniformity breaks and causes the initiation of instability.

## Additional Information

**How to cite this article**: Jalil Razavi, M. *et al.* Cortical Folding Pattern and its Consistency Induced by Biological Growth. *Sci. Rep.* **5**, 14477; doi: 10.1038/srep14477 (2015).
